# Do foot orthoses change lower limb muscle activity in people with flat-arched feet towards a pattern observed in those with normal-arched feet?

**DOI:** 10.1186/1757-1146-4-S1-O33

**Published:** 2011-05-20

**Authors:** George S Murley, Karl B Landorf, Adam R Bird, Hylton B Menz

**Affiliations:** 1 Department of Podiatry, Faculty of Health Sciences, La Trobe University, Bundoora, VIC, 3086, Australia; 2 Musculoskeletal Research Centre, Faculty of Health Sciences, La Trobe University, Bundoora, VIC, 3086, Australia

## Background

Various foot orthoses have been shown to alter tibialis anterior and peroneus longus electromyographic (EMG) muscle activity during walking. However, it is unclear whether these changes represent optimisation in muscle function. Therefore, two studies were conducted to determine; (i) whether people with flat-arched feet display altered muscle activity compared to those with normal-arched feet during gait and (ii) whether foot orthoses change muscle activity in people with flat-arched feet towards a pattern observed in those with normal-arched feet.

## Methods

A screening protocol that included clinical and radiographic measures was used to recruit participants with normal-and flat-arched feet. Sixty adults aged 18 to 47 years qualified for this study. Of these, 30 had normal-arched feet (15 male and 15 female) and 30 had flat-arched feet (15 male and 15 female). Two different foot orthoses were dispensed to the participants with flat-arched feet: (i) a heat-moulded (modified) foam prefabricated foot orthosis and (ii) a 20-degree inverted-style customised foot orthosis. EMG activity was recorded while walking from tibialis posterior and peroneus longus via in-dwelling wire electrodes, and from tibialis anterior and medial gastrocnemius via surface electrodes.

## Results

During the contact phase of gait, the flat-arched group exhibited increased activity of tibialis anterior (19% increase) and decreased activity of peroneus longus (13% decrease). During midstance/propulsion, the flat-arched group exhibited increased activity of tibialis posterior (26% increase) and decreased activity of peroneus longus (14% decrease), compared with those with normal-arched feet. During the contact phase of gait, tibialis posterior EMG amplitude decreased significantly with the prefabricated orthosis (19% decrease) and the customised orthosis (12% decrease) compared to the shoe only condition (p < 0.008). In contrast, during the midstance/propulsive phase, peroneus longus EMG amplitude increased significantly with the prefabricated orthosis compared to the shoe-only and customised orthosis conditions (19% and 14% increase, respectively; p < 0.030).

## Conclusions

Both foot orthoses had a significant effect on EMG amplitude of tibialis posterior and peroneus longus, however only the modified prefabricated orthosis changed peroneus longus EMG amplitude towards a pattern observed with normal-arched feet. Otherwise, there were few differences found between the modified prefabricated and customised orthoses.

